# Thermodynamic Signatures of Weyl Fermions in NbP

**DOI:** 10.1038/s41598-018-38161-7

**Published:** 2019-02-14

**Authors:** K. A. Modic, Tobias Meng, Filip Ronning, Eric D. Bauer, Philip J. W. Moll, B. J. Ramshaw

**Affiliations:** 1 Max-Planck-Institute for Chemical Physics of Solids, Dresden, 01187 Germany; 2 Institut für Theoretische Physik, Technische Universität Dresden, 01062 Dresden, Germany; 3 Los Alamos National Laboratory, Los Alamos, NM 87545 USA; 4 Laboratory of Atomic and Solid State Physics, Cornell University, Ithaca, NY 14853 USA

## Abstract

We present a high magnetic field study of NbP—a member of the monopnictide Weyl semimetal (WSM) family. While the monoarsenides (NbAs and TaAs) have topologically distinct left and right-handed Weyl fermi surfaces, NbP is argued to be “topologically trivial” due to the fact that all pairs of Weyl nodes are encompassed by a single Fermi surface. We use torque magnetometry to measure the magnetic response of NbP up to 60 tesla and uncover a Berry paramagnetic response, characteristic of the topological Weyl nodes, across the entire field range. At the quantum limit B^*^ (≈32 T), *τ*/*B* experiences a change in slope when the chemical potential enters the last Landau level. Our calculations confirm that this magnetic response arises from band topology of the Weyl pocket, even though the Fermi surface encompasses both Weyl nodes at zero magnetic field. We also find that the magnetic field pulls the chemical potential to the chiral *n* = 0 Landau level in the quantum limit, providing a disorder-free way of accessing chiral Weyl fermions in systems that are “not quite” WSMs in zero magnetic field.

## Introduction

**Figure 1:**
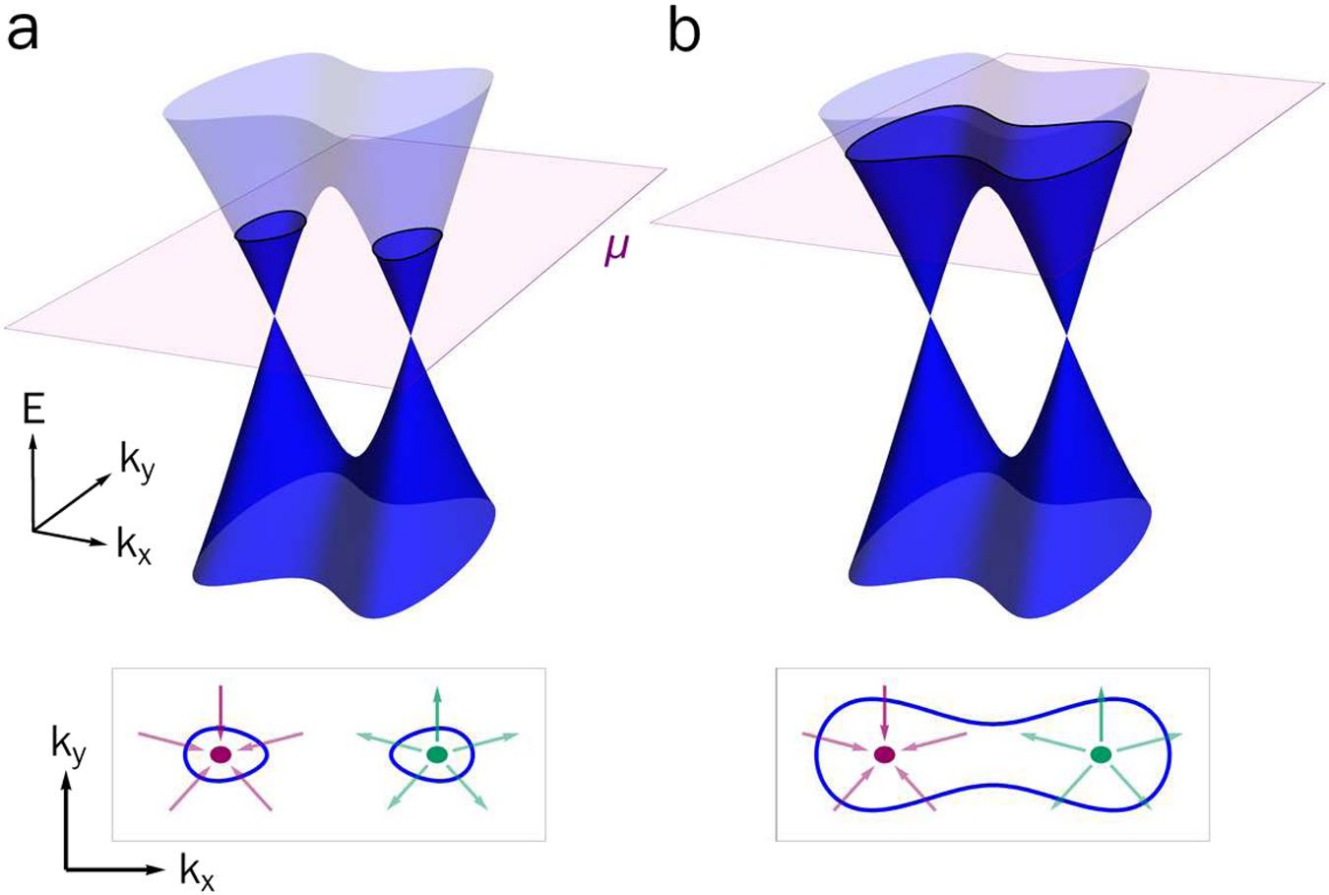
Schematic Fermi surface of a Weyl semimetal. With the chemical potential sufficiently close to the nodes (**a**), the left and right-handed Weyl nodes form separate chiral Fermi surfaces (green and purple arrows indicate sources and sinks of Berry flux). (**b**) When the chemical potential lies above the saddle point the two chiral Fermi surfaces merge into a single surface with no net Berry flux.

The Fermi surface topology of a WSM depends strongly on the position of the chemical potential. If it resides close to the band touching points (Weyl nodes), as it does in TaAs, separate Fermi surfaces of opposite chirality emerge, leading to novel phenomena such as the chiral magnetic effect^1–3^. If the chemical potential lies too far from the nodes, however, the chiral Fermi surfaces merge into a single large Fermi surface with zero Chern number and no net chirality. Figure 1 illustrates how the fine-tuning of energy scales determines the Fermi surface topology of a WSM.

Sensitivity to the fine-tuning of the chemical potential is prevalent in the inversion-symmetry broken Weyl-semimetals (Ta,Nb)(As,P)^4,5^. As the spin-orbit interaction in these materials is small compared to the overall electronic bandwidths, their Weyl nodes are not well-separated in momentum space and the energy barrier between the nodes is only a few 10s of meV. Photoemission experiments find evidence for Weyl Fermi-arcs in TaAs^6^ and NbAs^7^, indicating that the chemical potential is close to the nodes (Fig. 1a). Surface Fermi-arcs are also seen in the isovalent and isostructural phosphides^8^, however, the bulk chemical potential appears to be positioned such that the Weyl nodes are encompassed by a single Fermi surface^9,10^. With zero net quasiparticle chirality^11^, the phosphides have been argued to be intrinsically topologically trivial^10,12^ (Fig. 1b).

## Results

### Magnetic torque of NbP

**Figure 2:**
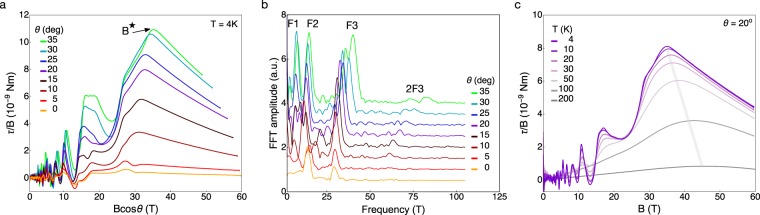
Magnetic torque of NbP. Panel (a) shows the angle dependence of *τ*/*B* versus *B* cos *θ* at *T* = 4 K. The angle *θ* is defined as the angle between the crystal *c*-axis and the applied magnetic field. We plot versus *B* cos *θ* to show that the quantum oscillation frequencies scale approximately as 1/cos *θ* at low angle, consistent with the roughly ellipsoidal Fermi surface geometry of NbP^10,16,30^. B^*^, the field at which the slope of *τ*/*B* changes sign, tracks the angle dependence of the quantum limit. (**b**) Fourier transform of the data in panel (**a**) with a polynomial background subtracted in the field range 1 ≤ *B* ≤ 30 T. Frequency components are labeled according to the notation used in^16^, and agree with previous studies^10,31^. (**c**) With field applied 20° away from the *c*-axis we measure the temperature dependence of *τ*/*B* from 4 to 200 K. B^*^ increases with increasing temperature (grey band) and is still visible even at 200 K. The positive sign of *τ*/*H* = (*χ*_*cc*_ − *χ*_*aa*_)*H* sin 2*θ* is consistent with a paramagnetic susceptibility and the known Fermi surface anisotropy of NbP^16^.

Here we show that NbP, despite the achiral character of its Fermi surface in zero field, exhibits signatures of the underlying topological band structure in the magnetic torque. Figure 2 shows the magnetic torque *τ* divided by magnetic field $(\vec{M}\times \vec{B}/|\vec{B}|)$—proportional to the magnetization—of NbP at multiple field orientations and temperatures. The magnitude of the background torque increases at low fields, overlayed by strong de Haas-van Alphen oscillations. At base temperature this behavior continues up to the field B^*^, at which point the torque exhibits a sharp kink and subsequent continual decrease in magnitude with increasing magnetic field. This behaviour is in contrast with the magnetization of a conventional semimetal such as graphite, which continues to increase with no change in slope at high magnetic fields^13^.

Torque measures the total magnetic anisotropy of the material, including the Landau diamagnetism from trivial pockets, Berry paramagnetism from Weyl pockets^14^, and core diamagnetism from the filled shells of Nb^3+^ and P^3−^ ^15^. NbP has four sections of Fermi surface: two nested sickle-shaped electron pockets (E1 and E2), and two nested banana-shaped hole pockets (H1 and H2) (following the notation of^16^). Both E1 and H1 encompass pairs of Weyl nodes, whereas E2 and H2 are trivial. Because each Fermi pocket is replicated symmetrically around the Brillouin zone such that time-reversal symmetry is preserved, torque disappears with field applied along the crystallographic directions. With the field applied slightly off the *c*-axis the quasiparticles form cyclotron orbits around the extremal orbits—“necks and bellies”—of all Fermi surfaces, giving rise to quantum oscillations with a frequency proportional to the cross-sectional area encompassed by the cyclotron orbits. In the notation of^16^, F1 corresponds to the *α*_2_ orbit on E2, F2 corresponds to the *β*_1_ orbit on E1, and F2 corresponds to the *α*_1_ orbit on E1.

Charge neutrality requires that the chemical potential move with magnetic field to keep the net carrier density—the number of electrons minus the number of holes—fixed. Magnetic fields introduce their own energy scale, the cyclotron energy, quantizing the energy spectrum into Landau levels (LLs). Above a threshold magnetic field, known as the quantum limit, only the *n* = 0 LL is crossed by the chemical potential. This is where the difference between trivial and Weyl electrons becomes important: the energy of the *n* = 0 LL for trivial pockets increases with magnetic field, while the *n* = 0 LL for Weyl pockets is field-independent. In NbP this field-independent *n* = 0 LL from the Weyl pockets acts as a sink for carriers from higher LLs and from the trivial pockets. At a large enough magnetic field the trivial pockets will become entirely depopulated^17^.

With field near the c-axis in NbP, all trivial Fermi surface cross-sections and the smaller orbits of the Weyl pockets enter their quantum limit below 32 tesla. This produces a complicated oscillation spectrum as the chemical potential adjusts with magnetic field, but ultimately the chemical potential ends up in the chiral *n* = 0 LLs of E1 and H1 at 32 tesla. The quantum limit of the largest cyclotron orbit coincides with the kink-field B^*^, and this field scale increases as the magnetic field is tilted away from the *c*-axis (Fig. 2a).

The magnetization is defined as the negative field-derivative of the free energy. The energy of the *n* = 0 LL in a trivial parabolic band increases linearly with magnetic field, dispersing as

1 $${E}_{0}=\frac{1}{2}\hslash \frac{eB}{{m}_{\perp }^{\ast }}+\frac{{\hslash }^{2}{k}_{z}^{2}}{2{m}_{z}^{\ast }},$$

where ${m}_{\perp }^{\ast }$ is the orbitally-averaged cyclotron mass perpendicular to the magnetic field, and ${m}_{z}^{\ast }$ and *k*_*z*_ are the mass and momentum along the field direction. In Weyl systems, on the other hand, each node has an *n* = 0 LL that is field independent and disperses linearly in *k*_*z*_:

2 $${E}_{0}=\pm \,\hslash {v}_{z}{k}_{z},$$

where *v*_*z*_ is the Fermi velocity along the field direction and the positive (negative) sign denotes the right (left) Weyl node. It was previously shown that Weyl and Dirac systems exhibit paramagnetism due to the field-independent *n* = 0 LL accepting an increasing number of carriers, leading to a net energy *decrease* with increasing magnetic field. This phenomenon, termed Berry paramagnetism, has been observed in the closely-related Weyl semimetal NbAs, where separate Fermi surfaces exist for left- and right-handed Weyl Fermions in zero magnetic field^14^. At the quantum limit, there is no longer a transfer of carriers from the *n* > 0 LLs to the *n* = 0 LL, leading to the observed abrupt change in slope of the magnetization at B^*^ and the subsequent decrease in magnetization with increasing magnetic field. This is qualitatively different from trivial systems, where the *n* = 0 LL is field dependent and the diamagnetic response continues to increase in the quantum limit.

The chemical potential in NbP lies above the saddle point separating the Weyl nodes at zero magnetic field (Fig. 1b). The fact that we observe a change in slope in *τ*/*B* at B^*^ signifies that the Weyl pockets dominate the magnetic response, and that the magnetic field shifts the chemical potential to the chiral *n* = 0 Landau levels above the quantum limit. We find that B^*^ is still visible at 200 K, but has shifted to higher magnetic field (Fig. 2c). We understand the increase in B^*^ with temperature as the thermal population of the diamagnetic *n* = 1 Landau level, which competes with the *n* = 0 Landau level, thereby requiring larger magnetic fields to observe the change in slope. The temperature dependence of B^*^ is also confirmed in our numerical calculations.

### Numerical simulation of magnetization in Weyl and trivial semimetals

We model our data using a time-reversal symmetric Weyl band structure whose chemical potential is above the saddle point in zero magnetic field. To analyze the dependence of the magnetization (∝ *τ*/*B*) on magnetic field strength and temperature, we employ a minimal tight-binding model with two atoms per unit cell^18^. Denoting the physical spin by *σ* and the sublattice pseudospin by *τ*, the zero-field Hamiltonian is given by $H={\sum }_{{\bf{k}}}\,{{\rm{\Psi }}}_{{\bf{k}}}^{\dagger }{ {\mathcal H} }_{{\bf{k}}}{{\rm{\Psi }}}_{{\bf{k}}}$ with

3 $$\begin{array}{rcl}{ {\mathcal H} }_{{\bf{k}}} & = & {\lambda }_{x}\,\sin ({k}_{x}a)\,{\sigma }_{x}{{\mathbb{1}}}_{\tau }+\sum _{j=y,z}\,{\lambda }_{yz}\,\sin ({k}_{j}a)\,{\sigma }_{j}{{\mathbb{1}}}_{\tau }\\  &  & +\,M(k)\,{\sigma }_{x}{\tau }_{x}-\mu \,{{\mathbb{1}}}_{\sigma }{{\mathbb{1}}}_{\tau },\end{array}$$

where ${{\rm{\Psi }}}_{{\bf{k}}}^{\dagger }=({c}_{{\bf{k}},\uparrow ,+}^{\dagger },{c}_{{\bf{k}},\downarrow ,+}^{\dagger },{c}_{{\bf{k}},\uparrow ,-}^{\dagger },{c}_{{\bf{k}},\downarrow ,-}^{\dagger })$ contains the creation operators for electrons of momentum *ħ***k** and spin *σ* = ↑, ↓ on sublattice *τ* = ±, and $M({\bf{k}})=m+m^{\prime} (2-\,\cos ({k}_{y}a)-\,\cos ({k}_{z}a))$ controls the separation of the Weyl nodes. In these expressions, *λ*_*x*_, *λ*_*yz*_, *m*, and *m*′ denote different hopping energies, *a* is the lattice constant, and *μ* is the chemical potential. This model has time-reversal symmetry ${\mathscr{T}}={\mathscr{K}}i{\sigma }_{y}{\tau }_{z}$ (${\mathscr{K}}$ denotes complex conjugation). In the remainder, we focus on the regime 2*m*′ + *m* > *λ*_*x*_ > *m* > 0, in which the low-energy physics is due to four Weyl nodes on the *k*_*x*_-axis. For ${\lambda }_{yz}\gg {\lambda }_{x}$, they are described by the expansion of the Hamiltonian to linear order in *k*_*y*_ and *k*_*z*_,

4 $$\begin{array}{rcl}{ {\mathcal H} }_{{\bf{k}}} & \approx  & {\lambda }_{x}\,\sin ({k}_{x}a)\,{\sigma }_{x}{{\mathbb{1}}}_{\tau }+m\,{\sigma }_{x}\,{\tau }_{x}\\  &  & +\,\sum _{j=y,z}\,{\lambda }_{yz}\,a\,{k}_{j}\,{\sigma }_{j}{{\mathbb{1}}}_{\tau }-\mu \,{{\mathbb{1}}}_{\sigma }{{\mathbb{1}}}_{\tau }\end{array}$$

We now calculate the Landau levels for a magnetic field applied along the *x*-axis (the field is measured in units of ${B}_{0}=\hslash {\lambda }_{x}^{2}/{\lambda }_{yz}^{2}{a}^{2}e$). The total free energy is then computed at fixed quasiparticle density by adjusting the chemical potential accordingly as a function of magnetic field. This corresponds to the physical situation in a three dimensional metal, where charge neutrality constrains the number of carriers and the chemical potential oscillates with magnetic field. The quasiparticle number is given as the number of occupied single-particle states above *E* = 0 in both time-reversal partner sectors combined, plus a constant corresponding to the filled valence band (this constant is independent of the magnetic field because the two time-reversal partner sectors have particle-hole symmetric spectra)^15^. While NbP contains 4 distinct Fermi surface pockets^16^, the constraint of fixed *net* carrier density must still hold. Our simple one-pocket model does not aim to reproduce the entire quantum oscillation spectrum, which is complicated due to the motion of the chemical potential and 4 Fermi surface pockets. Instead, we aim to capture the low-field Berry paramagnetism and the change in slope at the quantum limit when the chemical potential resides above the saddle point at zero magnetic field.

**Figure 3:**
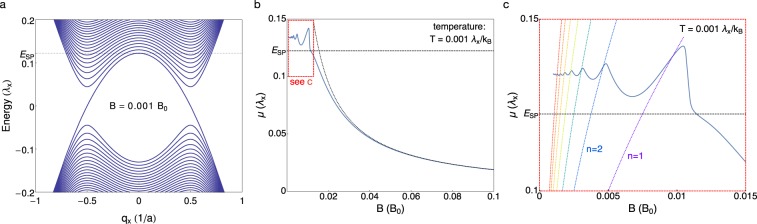
Landau levels for one sector and magnetic field dependence of the chemical potential. (**a**) Landau level spectrum of one of the two time-reversal partner sectors in our tight-binding model as a function of the (shifted) wave number *q*_*x*_ = *k*_*x*_ + *π*/2 for a magnetic field of amplitude *B* = 0.001*B*_0_ along the *x*-direction (where ${B}_{0}=\hslash {\lambda }_{x}^{2}/{\lambda }_{yz}^{2}{a}^{2}e$). At zero field, the model features Weyl nodes at *q*_*x*_ = ±0.5 and zero energy. The dotted horizontal line indicates the saddle point energy *E*_SP_. The spectrum of the time-reversal partner is particle-hole symmetric to this one. Panel (b) shows the field dependence of the chemical potential. At zero field, the Weyl nodes are located at zero energy, and the horizontal dashed line indicates *E*_SP_, the saddle point energy above which the Fermi surface encloses an equal number of nodes of each chirality (the topologically trivial regime). At large fields, the chemical potential is located within the *n* = 0 Landau level, and approaches *μ*(*B*) = *π*^2^*ρv*_*F*_/*B* (where *ρ* is the quasiparticle density, and *v*_*F*_ denotes the Fermi velocity, this is shown by the dotted line). Panel (c) is a zoom into the low-field region. The curved dotted lines depict the energies of the bottoms of the higher Landau levels with *n* = 1 through *n* = 7.

Figure 3c shows that the chemical potential is roughly constant at small fields, with quantum oscillations appearing as the chemical potential adjusts to keep carrier number fixed. Beyond B^*^, the field at which the *n* = 1 Landau level is depopulated at zero temperature, the large degeneracy of the *n* = 0 LL allows it to accommodate all quasiparticles. While the energy of the *n* = 0 LL is field-independent, its degeneracy increases linearly in *B*, pulling the chemical potential toward the Weyl nodes at *E* = 0 as *B* → ∞ (Fig. 3b). The computed magnetization, $M=-\,\frac{dF}{dB}$, for a single Weyl pocket agrees well with our observed data, confirming that the magnetic response of NbP is dominated by the Weyl pockets.

**Figure 4:**
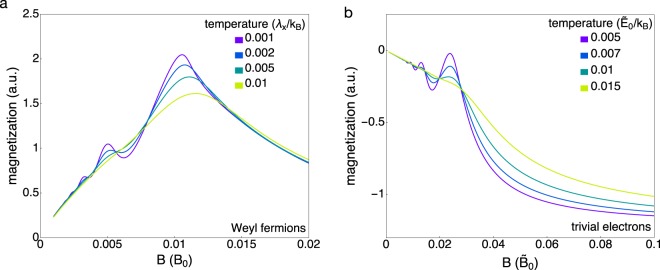
Simulated magnetization of Weyl Fermions and trivial quasiparticles. Panel (a) depicts the magnetization as a function of field for different temperatures. The magnetization (which is proportional to *τ*/*B*) increases at low fields due to the paramagnetic contribution of the *n* = 0 Landau levels, and then decreases at the quantum limit when there are no more *n* > 0 LLs to depopulate. Panel (b) contrasts this with the diamagnetic response of trivial Landau levels, which approaches a finite and constant value at large fields (here, magnetic fields are measured in units of ${\tilde{B}}_{0}=2{\tilde{E}}_{0}{m}_{\perp }/\hslash e$ and the chemical potential at zero field is $\mu =0.06\,{\tilde{E}}_{0}$, where *E*_0_ are units of energy).

This behavior can be contrasted to the magnetization of a free electron gas with parabolic dispersion. The large degeneracy of the LLs again implies that when the total carrier density is held constant, all quasiparticles bunch at the bottom of the *n* = 0 LL at large fields. Because the energies of all parabolic LLs (including the *n* = 0 LL) increase with field, however, the energy density is now asymptotically given by $\epsilon =\rho \hslash {\omega }_{c}/2$, where ${\omega }_{c}=\frac{eB}{{m}^{\ast }}$ is the cyclotron frequency and *ρ* is the quasiparticle density. In stark contrast to the experimentally observed behavior in NbP, the large-field magnetization for a trivial band approaches a constant finite value (Fig. 4b). The lack of slope change in the magnetization at the quantum limit, and the beginning of the saturation at high field, has been observed in the conventional semimetal graphite^13^.

## Discussion

Weyl Fermions exhibit unusual transport and optical properties due to the chiral anomaly^1–3,17,19^ and the mixed axial-gravitational anomaly^19,20^, and are the starting point for several predicted chiral states of matter^21–24^. Metals hosting Weyl nodes ought to be relatively common: in three dimensions the band crossings that give rise to linear energy-momentum dispersions do not require the fine tuning that they require in two dimensions^25,26^. Like NbP, several candidate materials have the chemical potential crossing far from the Weyl nodes forming topologically trivial Fermi surfaces in zero magnetic field. We have shown that direct experimental signatures of the Weyl nodes are visible in thermodynamic measurements, even when the Weyl nodes are encompassed by a single achiral Fermi surface. In addition, we demonstrate the use of high magnetic fields to shift the chemical potential to the fully chiral *n* = 0 Landau level in the quantum limit, greatly broadening the number of systems where Weyl fermions can be studied in practice. The same high magnetic fields also increase the Coulomb interaction between quasiparticles (generally weak in semimetals due to their high Fermi velocities), driving these systems closer to new symmetry-breaking and interacting states of matter such as chiral excitonic and density wave phases^21,27^, Luttinger liquids^24^, and unconventional collective excitations^28^.

## Methods

A single crystal of NbP was oriented on a Seiko Instruments peizoresistive microcantilever. The cantilever was incorporated into a balanced Wheatstone bridge circuit and 500 *μA* of excitation current was applied at 297.5 kHz.

Torque was measured as a function of magnetic field in the 65 tesla short-pulse magnet at the National High Magnetic Field Lab—Pulsed Field Facility in Los Alamos, New Mexico. A rotating sample stage was used to change the field-angle, and a pickup coil mounted on the sample platform was used to measure the angle. Measurements at 4 kelvin were performed with the sample in liquid helium; measurements above 4 kelvin were performed in helium exchange gas.

Magnetic torque on the sample bends the piezoresistive cantilever, unbalancing the circuit and producing a voltage proportional to the torque. This signal is converted to units of N.m via

5 $$4\frac{{\rm{\Delta }}V}{V}=\frac{{\pi }_{L}6\tau }{2w{t}^{2}},$$

where Δ*V* is the signal when the bridge becomes unbalanced, *V* is the excitation voltage, *π*_*L*_ is the piezoresistive coefficient of the cantilever, *w* is the lever width, *t* is the lever thickness, and *τ* is the torque^29^.

## Data Availability

All data is available upon request.
