# Author Correction: Thermodynamic Signatures of Weyl Fermions in NbP

**DOI:** 10.1038/s41598-020-59135-8

**Published:** 2020-02-06

**Authors:** K. A. Modic, Tobias Meng, Filip Ronning, Eric D. Bauer, Philip J. W. Moll, B. J. Ramshaw

**Affiliations:** 1 Max-Planck-Institute for Chemical Physics of Solids, Dresden, 01187 Germany; 2 Institut für Theoretische Physik, Technische Universität Dresden, 01062 Dresden, Germany; 3 Los Alamos National Laboratory, Los Alamos, NM 87545 USA; 4 Laboratory of Atomic and Solid State Physics, Cornell University, Ithaca, NY 14853 USA

Correction to: *Scientific Reports* 10.1038/s41598-018-38161-7, published online 14 February 2019

The Acknowledgements section in this Article is incomplete.

“The authors wish to thank A. Shekhter for useful discussions. B.J.R. acknowledges funding from LANL LDRD 20160616ECR ‘New States of Matter in Weyl Semimetals’, from the DOE-BES ‘Science of 100 Tesla’ program, and from the National Science Foundation under Grant No. 1752784. Work at Los Alamos National Laboratory was supported by the LDRD Program. T.M. is funded by Deutsche Forschungsgemeinschaft through GRK 1621, SFB 1143, and the Emmy-Noether program ME 4844/1. P.J.M. is supported by the Max-Planck-Society and the European Research Council (ERC) under the European Union’s Horizon 2020 research and innovation programme (grant agreement No. 715730).”

should read:

“The authors wish to thank A. Shekhter for useful discussions. B.J.R. acknowledges funding from LANL LDRD 20160616ECR ‘New States of Matter in Weyl Semimetals’, from the DOE-BES ‘Science of 100 Tesla’ program, and from the National Science Foundation under Grant No. 1752784. Work at Los Alamos National Laboratory was supported by the LDRD Program. T.M. is funded by Deutsche Forschungsgemeinschaft through GRK 1621, SFB 1143, and the Emmy-Noether program ME 4844/1. P.J.M. is supported by the Max-Planck-Society and the European Research Council (ERC) under the European Union’s Horizon 2020 research and innovation programme (grant agreement No. 715730). A portion of this work was performed at the National High Magnetic Field Laboratory, which is supported by the National Science Foundation Cooperative Agreement No. DMR-1157490, the State of Florida and the United States Department of Energy.”

